# Salt stress in the renal tubules is linked to TAL-specific expression of uromodulin and an upregulation of heat shock genes

**DOI:** 10.1152/physiolgenomics.00057.2018

**Published:** 2018-09-14

**Authors:** Lesley A. Graham, Alisha Aman, Desmond D. Campbell, Julian Augley, Delyth Graham, Martin W. McBride, Niall J. Fraser, Nicholas R. Ferreri, Anna F. Dominiczak, Sandosh Padmanabhan

**Affiliations:** ^1^The British Heart Foundation Centre of Excellence, Institute of Cardiovascular and Medical Sciences, College of Medical, Veterinary, and Life Sciences, University of Glasgow, Glasgow, United Kingdom; ^2^Wolfson Wohl Cancer Research Centre, Glasgow Polyomics, University of Glasgow, Bearsden, United Kingdom; ^3^University of Dundee, Ninewells Hospital, Dundee, United Kingdom; ^4^Department of Pharmacology, New York Medical College, Valhalla, New York

**Keywords:** heat shock, RNA-Seq, salt stress, sodium, thick ascending limb of the loop of Henle, uromodulin

## Abstract

Previously, our comprehensive cardiovascular characterization study validated Uromodulin as a blood pressure gene. Uromodulin is a glycoprotein exclusively synthesized at the thick ascending limb of the loop of Henle and is encoded by the *Umod* gene. *Umod^−/−^* mice have significantly lower blood pressure than *Umod^+/+^* mice, are resistant to salt-induced changes in blood pressure, and show a leftward shift in pressure-natriuresis curves reflecting changes of sodium reabsorption. Salt stress triggers transcription factors and genes that alter renal sodium reabsorption. To date there are no studies on renal transcriptome responses to salt stress. Here we aimed use RNA-Seq to delineate salt stress pathways in tubules isolated from *Umod^+/+^* mice (a model of sodium retention) and *Umod^−/−^* mice (a model of sodium depletion) ± 300 mosmol sodium chloride (*n* = 3 per group). In response to salt stress, the tubules of *Umod^+/+^* mice displayed an upregulation of heat shock transcripts. The greatest changes occurred in the expression of: *Hspa1a* (Log2 fold change 4.35, *P* = 2.48 e^−12^) and *Hspa1b* (Log2 fold change 4.05, *P* = 2.48 e^−12^). This response was absent in tubules of *Umod^−/−^* mice. Interestingly, seven of the genes discordantly expressed in the *Umod^−/−^* tubules were electrolyte transporters. Our results are the first to show that salt stress in renal tubules alters the transcriptome, increasing the expression of heat shock genes. This direction of effect in *Umod^+/+^* tubules suggest the difference is due to the presence of *Umod* facilitating greater sodium entry into the tubule cell reflecting a specific response to salt stress.

## INTRODUCTION

Blood pressure (BP) responses to salt intake are variable (50); however, epidemiologic, interventional, evolutionary and genetic studies in humans and animals have clearly demonstrated a link between salt intake and hypertension (HTN) (1, 2, 4, 7, 9, 10, 14, 22, 30, 40, 42, 51, 52). High salt sensitivity is estimated to be present in 51% of hypertensive and 26% of normotensive populations (20, 21). Although salt sensitivity is a well-recognized phenomenon in experimental and human HTN, the pathophysiological mechanisms are not fully elucidated (3). Recent systems approaches by means of metabolomics (31, 45) and proteomics (17, 32) have identified putative pathways that are affected by salt stress in plants and rats but to date studies in humans are lacking.

Osmoregulation, the cellular response to environmental changes of osmolarity and ionic strength, is important for the survival of living organisms. Large fluctuations in environmental osmolarity due to dietary salt may modulate stress responses across the nephron, affecting BP control. A family of proteins called heat shock factors (HSFs) are activated by the nonnative proteins that accumulate in response to salt stress. Activated HSFs binds to heat shock elements and stimulate transcription of heat shock protein (HSP)70. The promoter of HSP70 is stimulated in response to hypertonicity due to the tonicity-responsive enhancer binding protein (TonEBP) binding. TonEBP, also known as nuclear factor of activated T cells 5 (NFAT5), is the master transcriptional regulator for the cellular accumulation of organic osmolytes in the renal medulla (34, 53). Once NFAT5 is activated by hyperosmotic stress it triggers increased expression of osmosensitive genes that alter Na^+^ reabsorption in the thick ascending limb of the loop of Henle (TAL) (18). The rate of NaCl transport in the TAL is an important determinant of medullary hypertonicity, occurs via the Na^+^/K^+^/Cl^-^ cotransporter (Nkcc2) a protein expressed exclusively in the apical membrane of the TAL and macula densa cells (13). Nkcc2 and NFAT5 are sequentially expressed in the TAL, and inhibition of Nkcc2 activity by furosemide reduces expression of NFAT5 and its target genes in the renal medulla (43), implicating an important functional link between Nkcc2, NFAT5, and Na^+^ reabsorption. Hao et al. (18, 19) reported the Nkcc2 A isoform (Nkcc2A) contributes to the regulation of NFAT5 in primary cultures of medullary TAL cells exposed to hypertonic NaCl concentration.

Uromodulin (Umod) is a kidney protein exclusively synthesized at the TAL and is encoded by the *Umod* gene. We have reported previously that altered *Umod* expression is causal of HTN (37) and have since validated *Umod* as a blood pressure gene in a comprehensive characterization study in *Umod−/−* mice (16). *Umod^−/−^* mice display augmented Na^+^ excretion, thought to be a consequence of reduced expression of Nkcc2. This modulated Na^+^ reabsorption by reduced Nkcc2 leads to exaggerated natriuresis and lower arterial pressure in the *Umod^−/−^* mice; furthermore, these mice are not sensitive to salt-induced changes in BP consistent with findings in humans with salt-wasting phenotypes and hypotension (41, 44, 49). In a complimentary set of experiments, Trudu et al. (47) demonstrated that uromodulin-transgenic mice overexpressing *Umod* manifested salt-sensitive HTN, due to activation of the SPAK kinase and activating NH_2_-terminal phosphorylation of Nkcc2. These studies combined imply a permissive role of *Umod* in the modulation of Na^+^ transport. The specific pathways involved in salt tolerance in the TAL are not well defined, and to date there are no studies on transcriptome response(s) in the TAL to salt stress. Here we aimed to delineate salt stress pathways in TAL tubules to determine the changes in the TAL transcriptome associated with the expression of *Umod* and salt status.

## METHODS

### 

#### Experimental animals.

**Fig. 1.**
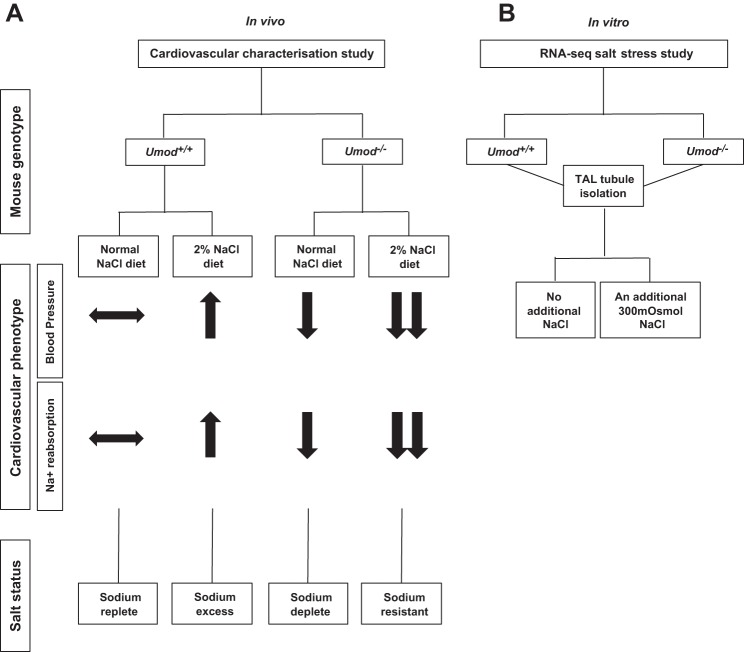
Study design. Our initial cardiovascular characterization studies in *Umod^+/+^* and *Umod^−/−^* mice have shown that *Umod^−/−^* mice have reduced systolic blood pressure, have limited sodium reabsorption at the thick ascending limb of the loop of Henle (TAL), and are not sensitive to dietary salt intake (2% NaCl in the drinking water for 6 wk) (16). The phenotype of each mouse strain is illustrated in (*A*). Salt status of *Umod^+/+^* and *Umod^−/−^* (in vivo) are as follows: *Umod^+/+^* (normal salt diet) represent salt repletion; *Umod*^+/+^ under salt-loading conditions (2% NaCl in the drinking water for 6 wk) represent a model of excess salt reabsorption; *Umod^−/−^* mice (normal salt diet) represent a sodium deplete model; and *Umod^−/−^* mice under salt-loading conditions (2% NaCl in the drinking water for 6 wk) represent a model of salt resistance. Therefore, due to the salt status and cardiovascular phenotype of each mouse strain, the current study utilized TAL tubules from each mouse under normal dietary conditions to take forward for in vitro salt stress studies. *B*: TAL tubules were isolated from both *Umod^+/+^* and *Umod^−/−^* mice and incubated in growth media for 4 h at 37°C under the following salt stress conditions: either incubation media supplemented with an additional 300 mosmol NaCl (salt stress group) or received no additional NaCl treatment, normal incubation media (control group) (*n* = 3 per group). Transcriptomic profiles using RNA-Seq were then determined in isolated TAL tubules in *Umod^+/+^* and *Umod^−/−^* mice (±300 mosmol NaCl). *n* = 3 biological reps per group. Male *Umod^+/+^* and *Umod^−/−^* mice of 5–7 wk of age were utilized in this in vitro study.

The *Umod^−/−^* mouse model was generated by Bates et al. (5) (Oklahoma University) along with the *Umod^+/+^* strain and used throughout this study. These mice have been maintained as breeding colonies at Glasgow University since 2010. The mice were housed under controlled environmental conditions, fed standard rat chow (rat and mouse No. 1 maintenance diet, Special Diet Services containing 0.19% Na^+^ and 0.32% Cl^−^), and water provided ad libitum. All animal procedures performed were approved by the Home Office according to regulations regarding experiments with animals in the United Kingdom. The genotype of the *Umod^+/+^* and *Umod^−/−^* mice was verified by end point PCR of tail genomic DNA with specific primers (forward: 5′ AGGGCTTTACAGGGGATGGTTG-3′ and reverse: 5′ GATTGCACTCAGGGGGCTCTGT 3′). Male mice of both strains were used in this study the experimental design is outlined in Fig. 1.

#### Isolation of TAL tubules.

Male *Umod^+/+^* and *Umod^−/−^* mice of 5–7 wk old were used for medullary TAL tubule isolation. Isolations were performed as previously described (12). In brief, mice were anesthetized with isoflurane, and the kidneys were perfused with sterile 0.9% saline solution via retrograde perfusion of the aorta. Once kidneys were excised they were cut along the corticopapillary axis, to expose the medulla. The inner strip of the outer medulla was dissected out and minced with a sterile blade in 0.1% (wt/vol) collagenase solution [collagen type IV collagenase prepared in Hanks’ balanced salt solution (HBSS) (Sigma Aldrich, Poole, UK and GIBCO, Paisley, UK)] that was gassed with 95% oxygen. The pulp was incubated for 10 min at 37°C. The cell suspension was sedimented on ice and mixed with HBSS containing 2% (wt/vol) BSA, and the crude suspension of tubules was collected. The remaining undigested tissue was collagenase treated a further three times. The combined supernatants were spun at a low speed (1,000 RPM) for 10 min and resuspended in HBSS. The resuspension was passed over a 52 µm nylon mesh membrane (Fisher Scientific, Loughborough, UK). The filtered solution was discarded, and the tubules collected on the mesh were washed with HBSS and centrifuged for 5 min at a low speed (500 RPM). The supernatant was aspirated, and the tubules were resuspended with Clonetics REGM growth media supplemented with the growth factor BulletKit (CC-3190): 0.5 ml hEGF, 0.5 ml hydrocortisone, 0.5 ml epinephrine, 0.5 ml insulin, 0.5 ml triiodothyronine, 0.5 ml transferrin, 0.5 ml GA-1000, 2.5 ml FBS. The tubule suspension was split into two separate 15 ml Falcon tubes, and the media was supplemented with an additional 300 mosmol NaCl (salt stress group) or received no NaCl treatment (control group) (*n* = 3 per group). The tubule suspensions were rotated for 4 h at 37°C. After the incubation period, the suspensions were centrifuged at a low speed (1,000 RPM) for 10 min, the supernatant removed, and the tubule pellets were taken forward for RNA isolation.

#### RNA extraction, quantification, and analysis.

Total RNA was extracted using Qiagen column based miRNeasy Mini kits (QIAGEN, Manchester, UK). Tubules were homogenized with QIAzol homogenization reagent included in the kit. RNA was eluted in 30 μl RNase-free H_2_O. A NanoDrop ND-1000 spectrophotometer (Thermo Scientific, Loughborough, UK) was used to measure RNA concentrations. This method is sensitive at measuring concentrations between 2 and 37,000 ng/μl of double-stranded DNA. Absorbance ratios (260 nm/280 nm) of ~2.0 for RNA indicated that the nucleic acid preparations were sufficiently free from protein contamination for downstream experiments. Additionally, the ratio of absorption at 260 nm and 230 nm was used as an indicator of RNA purity; pure RNA has a ratio of 2.0–2.2. The concentration of the sample is calculated with the Beer-Lambert Law of absorption; Concentration of RNA (μg/ml) = (A260 reading – A320 reading) × 40. RNA samples were stored at −80°C until time of RNA-Seq. Total RNA isolated was quality tested for degradation before RNA-Seq, via electrophoresis on the Agilent Bioanalyzer 2100 and a Eukaryote Total RNA Nano Series II chip. The analysis was run at the Molecular Biology Support Unit at the University of Glasgow. Electrograms produced indicate defined bands for 18S and 28S ribosomal RNA (rRNA) species and an RNA integrity number.

#### RNA-Seq data set of renal TAL tubules from mice exposed to no salt and salt stress (+300 mosmol).

Total RNA (500 ng) was isolated, and RNA-Seq performed using the NextSeq 500 platform operating 2*75 bp paired end cycles generating ~42 million reads per sample. Raw read files were adapter trimmed and quality-filtered using Cutadapt, to produce reads with mean quality score no less than 20, using Sanger quality scores. Read counts were obtained with Kallisto version 0.42.3 to pseudoalign reads to the GRCm38 (mm10) transcriptome. RNA-Seq data sets of all samples exhibited similar distribution and quantity of read counts.

#### Gene expression studies.

Quantitative real-time polymerase chain reaction (qRT-PCR) of total RNA extracted from TAL tubule isolations from male *Umod^+/+^ and Umod^−/−^* mice of 7 wk old was performed with Qiagen column based miRNeasy Mini kits (QIAGEN). Tubules were homogenized with QIAzol homogenization reagent included in the kit. cDNA was prepared using Applied Biosystems TaqMan Reverse Transcription Reagents. We utilized 1 μg of total RNA template for downstream applications. Determination of mRNA abundance of specific genes was assessed by qRT-PCR (ThermoFisher, Paisley, UK); *n* = 3 tubule isolations per group (no additional NaCl vs. +300 mosmol NaCl). Results were normalized to the housekeeper Gapdh (Mm03302249). Gene expression probes used: Mouse TaqMan probe Hspa1b (Mm03038954_s1), Mouse TaqMan probe Dnajb1 (Mm00444519_m1), and Mouse TaqMan probe Nr0b2 (Mm00442278_m1) (ThermoFisher, Paisley, UK).

#### Statistical analysis.

RNA-Seq analysis of differentially expressed genes (DEG) was conducted with the Bioconductor package DESeq2 (Stanford, CA) (28). DESeq2 assumes that the RNA-Seq counts are negative binomial distribution. DESeq2 utilizes generalized linear modeling and variance-reduction techniques for estimated coefficients to test individual null hypotheses of zero log2 fold changes between high salt and low salt for each gene. It uses both an independent filtering method and the Benjamini-Hochberg procedure to improve power and control the false discovery rate (FDR). The default DESeq2 options were used, and an FDR Q value < 0.1 threshold for *Umod^+/+^* mice and FDR Q value < 0.05 for *Umod^−/−^* mice were used to identify DEG.

We performed an ontology enrichment analysis looking for differentially expressed mRNA levels in each of the 2,813 gene-sets for *Umod^+/+^* and 2,352 gene-sets for *Umod^−/−^* mice of Gene Ontology (GO) for *Mus musculus,* which was obtained with the R package BiomaRt (48). For this we used the R package *Piano* (48), which enables functional characterization and interpretation through gene set analysis (GSA). We used *Piano* as recommended in the authors’ paper. In brief, *Piano* implements 12 GSA methods, taking as input either *P* value plus direction of effect, or *t* score, per gene and the fold change. Of these 24 combinations of the enrichment analysis method with the input statistic type, 16 are valid. For each GO term, a *P* value for enrichment was obtained via each of these 16 method/input-type combinations. These *P* values were estimated using 100,000 gene sampling permutations. For each of the 16 method/input-type combinations, the GO terms were ranked according to *P* value. The “consensus score” for each GO term was the median of these 16 ranks for the corresponding GO term. The “median *P* value” for each GO term was the median of the 16 corresponding *P* values. Furthermore, the above was done for five different enrichment hypotheses. The central hypothesis tested (“nondirectional”) was whether a GO term is enriched for differentially expressed genes. Two further hypotheses concerned upregulation *1*) enrichment for upregulation, ignoring any downregulation (“mixed-directional”) and *2*) enrichment for upregulation, penalized by any downregulation (“distinct-directional”). Two corresponding downregulation hypotheses were also tested.

## RESULTS

The study design is described in Fig. 1. Previously we have performed cardiovascular characterization studies in *Umod^+/+^* and *Umod^−/−^* mice (±2% NaCl in the drinking water for 6 wk). These studies have demonstrated that *Umod^+/+^* mice are a model of sodium retention, and *Umod^−/−^* mice are a model of sodium depletion (Fig. 1*A*). Based on sodium status of each strain, the present study utilized RNA-Seq technology to investigate the specific effects of TAL salt stress in vitro depending on *Umod* expression (Fig. 1*B*).

### 

#### Salt-related DEGs in TAL tubules.

**Fig. 2.**
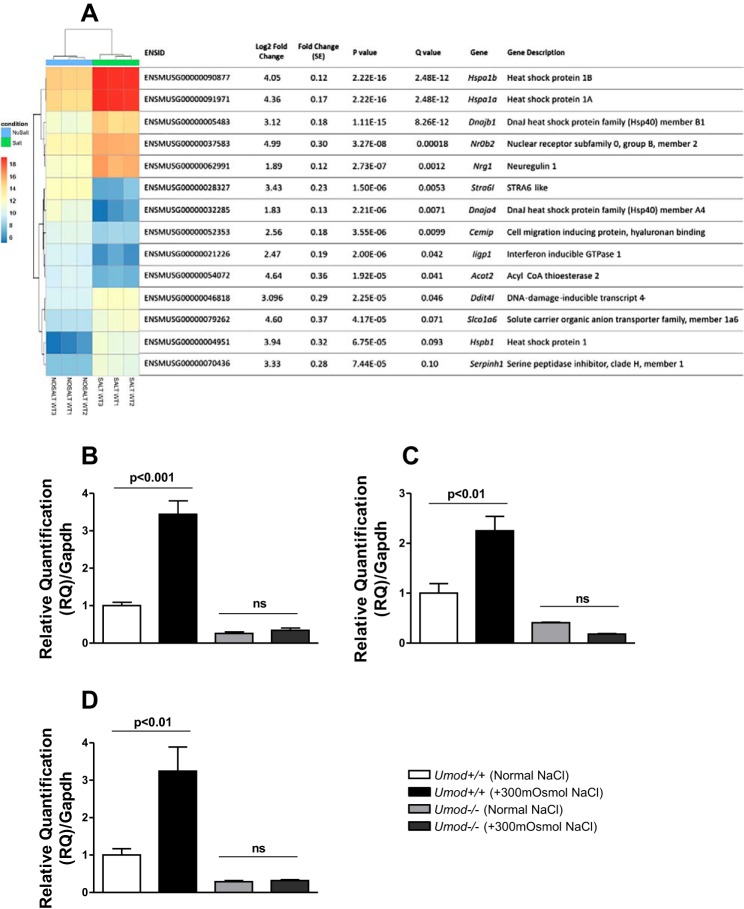
Heat-map visualization of RNA-Seq transcriptome analysis of transcription levels for the most significantly differentially expressed genes in *Umod^+/+^* mice TAL tubules (±300 mosmol NaCl). *A*: the heat map shows differentially expressed genes (DEG) significant for one pairwise test. Analysis was performed using DESeq2 with the read counts as input. The heat map indicates the genes with significantly altered expression [false discovery rate (FDR) Q value <0.1]. The colors indicate the transform counts as log2(*x* + 1), where *x* = read counts, with the red spectra indicating high expression and the blue indicating low expression. Gene details are depicted on the *right* along the vertical axis. NOSALTWT1, NOSALTWT2, and NOSALTWT3 are the control group (no additional salt treatment added to the incubation media) and SALTWT1, SALTWT2, SALTWT3 are the tubules treated with additional 300 mosmol NaCl in the incubation. Fold change is reported as the log2 value with its standard error of estimation. The final *P* values after false discovery rate correction is given as the Q values. Quantitative (q)RT-PCR analysis show an increased expression of Hspa1b (*B*), Dnajb1 (*C*), and Nr0b2 (*D*) in *Umod^+/+^* TAL tubules when treated with an additional 300 mosmol NaCl. Data are shown as mean RQ ± SE (Cycle threshold values were normalized to Gapdh mRNA and expressed relative to *Umod^+/+^* tubule isolations under normal NaCl conditions), *n* = 3 per group. Analyzed by one-way ANOVA followed by Tukey’s post hoc multiple comparison test. ns, Not significant. *P* < 0.05 is deemed statistically significant.

In *Umod^+/+^* mice tubules, of the 43,629 transcripts detected, DESeq2 filtered and analyzed 22,355 transcripts, out of which 14 DEGs were identified with a nominal significant difference in gene expression (FDR Q value <0.1; 5 decreased and 9 increased) (Fig. 2). In *Umod^−/−^* mice tubules, of the 43,629 transcripts detected, DESeq2 filtered and analyzed 15,723 transcripts. Of the DEGs, 178 were identified with a nominal significant difference in gene expression (FDR Q value <0.05; 27 decreased and 151 increased; data not shown).

In *Umod^+/+^* mice, the transcript count for *Umod* (ENSMUSG00000030963) were 538,475; 498,026; 777,919 for +300 mosm NaCl *Umod^+/+^* mice, and 353,713; 425,034; 256,762 for no added NaCl *Umod^+/+^* mice. The log2 fold change for *Umod* was 1.10 with an FDR corrected *P* value of 0.17. In contrast, the *Umod^−/−^* mice showed lower *Umod* transcript counts: 7,756; 6,951; 9,533 for +300 mosm NaCl *Umod^−/−^* mice, and 9,920; 8,916; 6,782 for no added NaCl *Umod^−/−^* mice. The log2 fold change for *Umod* in this data set was 0.03 with an FDR corrected *P* value of 0.8.

In *Umod^+/+^* mice, data analysis for biological interpretation of DEGs revealed among the upregulated genes a cluster of transcripts involved in heat stress (*Hspa1b, Hspa1a, Dnajb1, Dnaja4*) (Fig. 2), while the downregulated genes did not demonstrate any obvious enrichment for pathways. qRT-PCR validated the overexpression of *Hspa1b, Dnajb1*, and *Nr0b2* (Fig. 2). In the *Umod^−/−^* mice, DEGs revealed a multitude of upregulated genes across a range of pathways, but no single predominantly enriched pathway (data not shown).

**Fig. 3.**
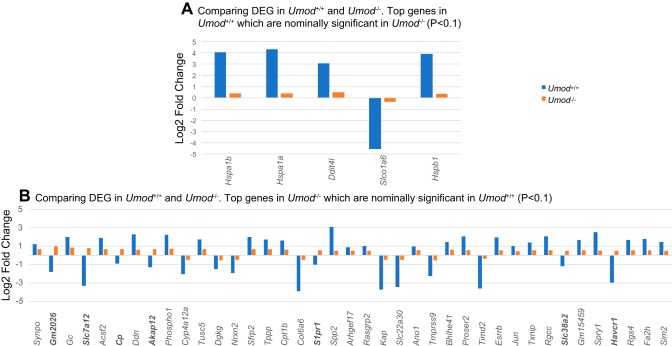
Nominally significant genes in either *Umod^+/+^* or *Umod^−/−^* isolated TAL tubules (+300 mosmol NaCl). *A*: comparison of the log2 fold change of the genes that are highly significant in *Umod^+/+^* mouse TAL tubules (q < 0.1) and nominally significant in *Umod^−/−^* mouse TAL tubules (*P* < 0.1); of these five significant genes, three belong to the heat shock protein family. *B*: comparison of the log2 fold change of the genes that are highly significant in *Umod^−/−^* mouse TAL tubules (q < 0.05) and nominally significant in *Umod^+/+^* mouse TAL tubules (*P* < 0.1); out of the 39 significant genes, 7 of them (in boldface) are discordant in their direction of expression in both the groups.

We compared the direction of effect of all the DEGs in tubules of *Umod^+/+^* and *Umod^−/−^* mice (summarized in Fig. 3). Genes significantly overexpressed in *Umod^−/−^* mouse TAL tubules showed log2 fold change >3, and the corresponding expression levels in *Umod^+/+^* mouse TAL tubules were substantially smaller, but the directions of effect were similar. Of the significant DEGs in *Umod^−/−^* mouse TAL tubules, gene expression levels were generally higher compared with *Umod^+/+^* mouse TAL tubules. However, seven out of the 39 (Q < 0.05) genes showed discordant directions of effect between *Umod^−/−^* and *Umod^+/+^* mouse TAL tubules: *Gm2026, Slc7a12, Cp, Akap12, S1pr1, Slc38a2,* and *Havcr1*.

#### Gene set analysis.

**Fig. 4.**
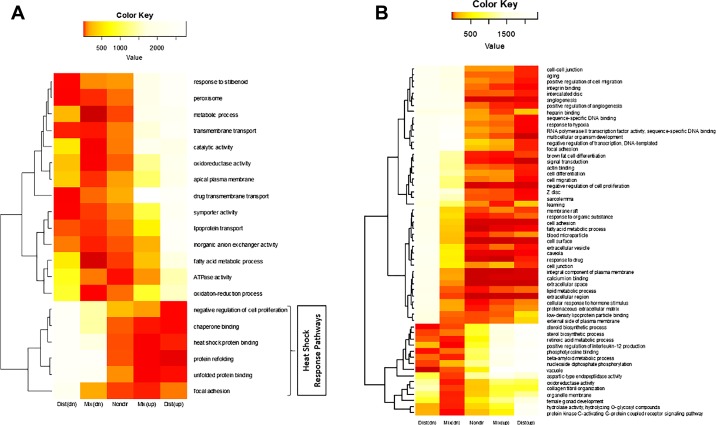
Pathway enrichment analysis of *Umod*^+/+^ (*A*) and *Umod*^−/−^ (*B*) mice TAL tubules postincubation with 300 mosmol NaCl. Enrichment was performed using *Piano* with 100,000 permutations across the gene sets and subsequently ranked across five hypotheses based on the differential expression. Consensus ranks are depicted as a heat map. Consensus scores and *P* values were calculated using the median values of the ranks per hypothesis. Three directional classes have been specified in the enrichment analysis: nondirectional, mixed-directional, and distinct-directional. In the nondirectional class, information on the direction of differential expression is removed. Mixed-directional class contains information indicating whether the gene set is significantly affected by the DEG in either or both directions; two *P* values are assigned for each gene set under this class, one for upregulation and the other for downregulation. The distinct-directional class identifies gene sets that are significantly altered by the DEG only in a particular direction; two *P* values are reported for each gene set, one for each direction. The consensus scores for heat shock binding protein and chaperone binding are very low and hence are ranked highest in nondirectional, mixed-directional up, and distinct-directional up, suggesting significant upregulation of the genes in those pathways under salt stress. The gradient of the heat map depicts the consensus scores, the red spectra indicating the lowest consensus scores.

Gene set analysis (GSA) was used to identify GO categories (of which there were a count of 2,813 gene-sets for *Umod^+/+^* mouse tubules and 2,352 gene-sets for *Umod^−/−^* mouse tubules) that were enriched for genes that were differentially expressed across treatments (no salt vs. +300 mosmol NaCl). This GSA was performed with the R package *Piano* (48). Among the most significantly upregulated categories were heat shock and chaperone binding (*Umod^+/+^* mouse tubules), while anion transporters predominate among the downregulated pathways in response to salt stress (*Umod^+/+^* mouse tubules, Fig. 4*A*). In *Umod^−/−^* mouse tubules, the range of upregulated and downregulated pathways are summarized in Fig. 4*B*.

## DISCUSSION

In this study, we show that significant overexpression of transcripts of heat shock genes (*Hspa1a* and *Hspa1b*, members of the HSP70 family, and *Dnajb1* and *Dnaja4*, members of the HSP40 family) is a specific response to salt stress in renal TAL cells. The unique aspect of this study is the use of *Umod^+/+^* vs. *Umod^−/−^* mouse tubules to differentiate the specific effect of salt at the TAL. *Umod^−/−^* are salt-resistant mice, and as *Umod* is exclusively expressed in the TAL our results demonstrate specific pathways stimulated in response to salt in the TAL. Our findings also provide insights into the evolutionary selection of *Umod* increasing ancestral allele in humans, with consequent salt-sensitive HTN becoming a maladaptive phenotype.

Salt stress is an unfavorable condition for many organisms and as a response, cells express HSPs to act as chaperones to perform a number of biological processes such as: transcription, translation and posttranslational adaptations, protein folding, protein targeting, and disaggregation of proteins. The HSP70 family proteins have been shown to play a substantial role in NaCl reabsorption in the medullary region of the kidney, and circulating HSPs have been associated with salt-sensitive HTN (38). The HSP40 family are cofactors for HSP70. HSP40/HSP70 complexes have been implicated during cellular stress by altering water and ion transport systems in the renal medulla leading to changes in tissue-specific gene expression profiles (6, 11, 26). HSPs act by capturing an unfolded polypeptide by recognizing exposed hydrophobic patches. This chaperone could be HSP70 itself or a J-domain cochaperone (HSP40) that forms a complex with HSP70. Cohen et al. (8) reported that salt-induced stress increased the mRNA expression of HSP70 in cultured Madin-Darby canine kidney cells, consistent with our findings here in tubules from *Umod^+/+^* mice. They postulated that HSP70 may play a role in the kidney to stabilize proteins in the face of the elevated and potentially denaturing ionic concentrations that accompany sudden changes in the medullary osmotic environment. Contrary to these findings Medina et al. (33) reported neither the HSP70 message nor its protein product differed significantly in medulla tubules, possibly due to prolonged water diuresis that might have attenuated expression of HSP70. Nevertheless, in the past few years it has become increasingly clear that HSPs may contribute decisively to survival of medulla cells by conferring protection against the extremely high interstitial solute concentrations (36).

Here, our novel transcriptomic findings suggest a biological link between salt stress in the TAL and interactions between *Umod* and HSPs. HSPs are involved in salt stress pathways to protect renal cells to maintain normal Na^+^ homeostasis at this nephron segment. We observed a striking difference in the transcriptomic profile in *Umod^−/−^* mice TAL tubules in response to salt with HSPs not featuring in the set of 178 genes attaining experiment-wide significance. The TAL tubules of *Umod^−/−^* mice showed a greater number of DEGs with substantially smaller effect sizes in terms of log2 fold change in contrast to that of tubules from *Umod^+/+^* mice. The direction of effect of the HSP in *Umod^+/+^* tubules suggests that the difference is due to the presence of *Umod* facilitating greater Na^+^ entry into the *Umod^+/+^* tubule cell and thus reflecting a specific response to salt stress. We and others have shown previously that *Umod* is associated with salt-sensitive HTN, and this is likely through Umod-Nkcc2 interaction (16, 47). The genes that attained statistical significance in the TAL tubules of *Umod^−/−^* mice represented a wide spectrum of pathways with no single pathway showing a predominant contribution. Many of the genes that appear elevated in the *Umod^−/−^* TAL tubules are electrolyte transporters and glomerular proteins: *Atp1a2* (ATPase, Na^+^/K^+^ transporting, alpha 2 polypeptide), *Aqp4* (aquaporin 4), *Slc12a1* (solute carrier family 12, member 1), *Kcnk3* (potassium channel, subfamily K, member 3), *Slc5a3* [solute carrier family 5 (inositol transporters), member 3], *Hsd17b11* [hydroxysteroid (17-beta) dehydrogenase 11], *Podxl* (podocalyxin-like), *Nphs1* (nephrosis 1, nephrin), *Syt7* (synaptotagmin VII), *Car3* (carbonic anhydrase 3), and *Cyp4a12a* (cytochrome P450, family 4, subfamily a, polypeptide 12a), indicating that these responses are related to increased luminal NaCl. Interestingly, *Slc12a1* which encodes Nkcc2, is significantly differentially overexpressed in *Umod^−/−^* mouse TAL tubules but shows no differential expression in *Umod^+/+^* mouse TAL tubules. It has been previously demonstrated that total Nkcc2 expression is increased in *Umod^−/−^* mice due to an abundance of “inactive” intracellular Nkcc2 expressed in the subapical vesicles (35). Using a less conservative significance threshold we find that 18% of the genes that are differentially expressed in *Umod^−/−^* mouse TAL tubules show an opposite direction of effect in *Umod^+/+^* mouse TAL tubules, but in general the majority of the genes show concordant direction of effect. *Umod* is coexpressed at the apical surface of the TAL with Nkcc2 and is reported to play a key role in Na^+^ homeostasis and BP control (16, 47). A positive correlation between urinary Umod and dietary salt intake revealed that in subjects with high salt sensitivity, there is a greater excretion of Umod in the urine compared low salt intake (46). Likewise, there is a direct relationship between high salt intake and *Umod* mRNA expression (16).

The accumulation of organic osmolytes is only one component of the adaptive process allowing medullary cells to survive in the harsh environment of salt stress. This effect is at least partially attributable to the chaperoning activities of HSP40/HSP70 complex (36). Ma et al. (29) have previously demonstrated the rescue of Umod to the apical surface of the TAL is mediated by the restoration of the level and subcellular localization of cytosolic chaperone HSP70 during salt stress. It seems likely we have identified a novel link between HSPs and salt stress pathways in the TAL, implicating potential biological mechanisms to be investigated in the management of salt-sensitive HTN. There are some limitations to the current study. We have not measured apoptosis in the TAL tubules following the salt stress conditions; however, we speculate HSP70 is part of a protective mechanism, which is not present or needed in the *Umod^−/−^* TAL. Future studies will focus on exacerbating apoptosis by blocking HSP70 and HSP pathways in TAL tubules of *Umod^+/+^* mice. This would allow the investigation of pathogenetic pathways that emerges from these studies.

In the context of human essential HTN, antibodies to HSP70 are commonly observed and polymorphisms in HSP70 gene expression have also been associated with HTN in specific populations (27, 39). Pons et al. (39) have shown that during salt stress, the increased expression of HSP70 in medulla region of the kidney causes an activation of T cells as a response, leading to salt-sensitive HTN. Pockley et al. (38) reported IgG anti-HSP70 antibodies in the plasma of hypertensive patients, which is in concordance with preliminary studies from Pons et al. (39). This prompted the exploration of the proliferative responses of peripheral blood lymphocytes in a small group of well-defined patients with essential HTN to the antigenic peptide used in their experimental studies (39). The selection of patients was designed to limit the age range and exclude conditions that are known to be associated with anti-HSP70 antibodies, and the results showed a clear separation between the proliferative response of the patients and the control subjects. These studies provide evidence that autoimmunity plays a role in salt-sensitive HTN and identifies HSP70 expressed in the kidney as one key antigen involved in the development of autoimmune reactivity in the kidney and thereby in the impairment of physiological mechanisms of sodium excretion that accompanies salt-sensitive HTN.

Genome-wide association studies in humans have shown single nucleotide polymorphisms in the promoter region of the *Umod* gene to be associated with renal function and HTN (23–25, 37). Specifically, the allele associated with higher *Umod* levels are associated with higher BP and lower estimated glomerular filtration rate. The results from this study indicate that the increase in BP in those with the *Umod*-increasing genotype may be influenced by multiple mechanisms including those mediated by activation of HSP. A recent study showed global frequencies of the *Umod* alleles significantly correlated with pathogen diversity and prevalence of antibiotic-resistant urinary tract infections (UTIs), but not with the latitudinal clines in the frequencies of variants associated with salt sensitivity (15). The implication of this study is that *Umod* ancestral allele has been kept at a high frequency because of its protective effect against UTIs and salt-sensitive HTN may be a maladaptive phenotype through increased sodium reabsorption. It is also possible that the *Umod*-increasing allele may have arisen to preserve sodium in the salt-poor evolutionary past. Our findings of activation of heat shock pathway in response to salt suggests that HSPs are activated as a protective measure against the influx of sodium into the tubule cells; however, there is evidence that they may also facilitate salt-sensitive HTN through other independent pathways and activate the immune system, which may protect against infections. Further work will focus on elucidating the role of *Umod* and HSPs on infection and salt sensitivity as these are critical to downstream efforts to modulate *Umod* for HTN treatment.

## GRANTS

This work was supported by the British Heart Foundation (PG/12/85/29925, S. Padmanabhan) and Wellcome Trust Institutional Strategic Support Fund Catalyst Grant (Project code 71559/1, L. A. Graham).

## DISCLOSURES

No conflicts of interest, financial or otherwise, are declared by the authors.

## AUTHOR CONTRIBUTIONS

L.A.G. and S.P. conceived and designed research; L.A.G. performed experiments; L.A.G., A.A., D.D.C., J.A., and S.P. analyzed data; L.A.G., N.J.F., N.R.F., and S.P. interpreted results of experiments; L.A.G., A.A., and S.P. prepared figures; L.A.G. drafted manuscript; L.A.G., D.G., M.W.M., N.J.F., N.R.F., A.F.D., and S.P. edited and revised manuscript; L.A.G., A.A., D.D.C., D.G., M.W.M., N.J.F., N.R.F., A.F.D., and S.P. approved final version of manuscript.
